# New Strategies and Practices of Design Education Under the Background of Artificial Intelligence Technology: Online Animation Design Studio

**DOI:** 10.3389/fpsyg.2022.767295

**Published:** 2022-05-16

**Authors:** Tianran Tang, Pengfei Li, Qiheng Tang

**Affiliations:** ^1^School of Artificial Intelligence, Dongguan Polytechnic, Dongguan, China; ^2^Arts College of Jinan University, Guangzhou, China; ^3^Faculty of Innovation and Design, City University of Macau, Macau, Macao SAR, China

**Keywords:** artificial intelligence, design education, design studio, online, deep learning, education strategy

## Abstract

This study is based on the background of how artificial intelligence (AI) technology is applied to the field of creativity and design education to improve the design vision, teaching methods, and actual design productivity of practitioners. The purpose of the research is to compare traditional design education and new design education methods combined with AI technology. Taking the Technological Pedagogical Content Knowledge (TPACK) technology integration model as the starting point, a comprehensive evaluation is selected for different types of research to explore the animation design professional courses in design education, the content of students’ perception preferences, and the evaluation of ease of learning so as to conduct research and analyze AI technology. Design new education strategies and practice methods under the background. In the research, a comparative experimental study was conducted on 40 first-year students majoring in animation design. The results show that through online design studio project practice, with personalized project learning guidance, the learning needs of students to show a better trend, and customized learning and project practice content can enhance the learning experience and performance of students. In the future, we can further expand the scope of analysis, include more case studies, and conduct more comprehensive research, including how to deal with the expansion of the platform for students’ learning of design in situations similar to coronavirus disease 2019 (COVID-19) that profoundly affects our lives, and how the project is applied in practice.

## Introduction

Artificial intelligence (AI) technology has a broad application prospect in the design field. With further penetration of big data technology, three-dimensional (3D) printing technology, virtual reality, and augmented reality technology (Oliver, 2003), digital technology, and design art are showing a trend of high integration. In China, some colleges and universities combine basic programming software, such as Processing and Arduino, with the teaching of design professional courses to deal with the combination of digital technology and design. With the in-depth application of AI technology, the design field will be driven to make greater changes in the artistic presentation of design. These changes not only require design practitioners and learners to face adaptation as educators also face technological changes at the level of design education. Challenges from learner behavior, teachers’ teaching methods, and teaching strategies, such as learning new digital skills and using AI technology in teaching, refer to the use of AI system suggestions. Therefore, in the context of AI technology, many key issues, such as the effectiveness of technical performance, learning pedagogy, user experience, learning environment, and interactive content, need to be resolved. Through an online design studio based on a mature design studio education strategy, the use of AI technology is used to carry out teaching activities and related design project practice and explore whether it can enhance the learning and design practice of design students. In order to assist educators in making decisions and adjusting teaching methods to solve problems, we should design a new strategy that adapts to new technologies and can specifically and effectively be implemented in education.

## Literature Review

In the field of art and design, the development of the times and the needs of society promote the continuous production of new types, styles, and branches in art design. At the same time, a series of cross-professional, multi-disciplinary, and major social and cultural themes have emerged. The comprehensive design has become a new “landscape” in the design field with its diversification of cultural elements, richness of structure, crossover, and comprehensiveness of forms and techniques, etc. (Jing, 2020). As early as 2003, the art experience of the traditional virtual art design and virtual reality art design has been demonstrated. Later, a comparative study of the traditional virtual art design and the current new media art design was conducted in terms of aesthetics and styles (Oliver, 2007). The evolution of interactive art design, intelligent art design, and creative ideology from 1964 to 2011 expands the role of intelligent factors in art design in the future. A 2016 portrait of Edmond Belamy created entirely by AI explores the relationship between art design and AI. Artists, such as Mario Klingemann, reconstructed artificial neural networks and used AI recognition technology to imitate many works of art masters to create a self-portrait called 79530 (Adhikari, 2016). In addition, there are many studios and laboratories, artists, designers, and artist groups dedicated to the research of future art and design, and they are carrying out AI art design practices. For example, the Australian artist Stelare is committed to the creative research of intelligent robot behavior art design, mainly combining intelligent robots and humans, and then controlling the artistic creation of human behavior. Are also engaged in the artistic creation of intelligent robot performance. In addition, Ahmed Elgammal, under the influence of AI technology, cloud computing, and big data background, thought about how to use AI technology to achieve high-quality and efficient visual design creative output. He also thought of how to use these technologies to help design a series of questions, finally how to achieve a more reasonable visual design and serve the purpose of a larger range of service objects to a greater extent, and thus launch a series of explorations (Archer, 1979; Arzarello et al., 2002; Archambault and Crippen, 2009; Doering et al., 2009; Comas-Quinn, 2011; Cowan, 2013; Baltacı, 2014; Fleischmann, 2015; Goertzel, 2015; Bagli and Serifoglu, 2021).

In the field of education, facing the ever-changing globalized world, it is necessary to constantly adjust and reflect on the mission and profession of teachers in accordance with the various new requirements and challenges. At the teacher level, the development of professional activities is affected by many factors, including the speed and depth of technological development. The development of technology will put forward new requirements for the roles of teachers in professional activities, requiring teachers to actively respond to external factors. Environment to achieve a role change (Chen et al., 2021). With the rapid development of a new generation of information technology represented by the Internet, big data, and the Internet of Things, international economics, technological and cultural exchanges, and cooperation tend to be more comprehensive and in-depth. In the context of this era, promoting the intersection and integration between design disciplines and other disciplines has become the main way for world-class universities to promote design innovation and cultivate high-quality compound innovative talents. This has created new challenges for the current teaching methods of teachers. Facing the higher demands of the development of the times, the current art design education has not really changed from “discipline-oriented” to “problem-oriented,” and the comprehensive art design education concept has not been implemented. Comprehensive curriculum construction and teaching design, system construction of comprehensive design education, diversified comprehensive talent training model, etc., still face many practical problems, which are worthy of further research and exploration. The research of AI art design theory mainly includes knowledge of the background of the AI art design, the relationship between AI technology and art design, and the artistic thinking of artists under the background of AI technology. However, similar research are often scattered. There is not a single book named after AI art design and there is no complete theoretical system for the research objects, characters, and methods of the AI art design. At the student level, it is believed that in universities, students can participate in scientific research under the guidance of professors, and thus obtain a lifelong subject thinking mode. As far as design education is concerned, the cultivation of the design thinking mode of the subject is particularly important. In addition, the integration of science and technology, art, and design makes the training goals of students need to be broader and targeted-oriented, data-tracking deep learning for students. Adaptive learning is also important for cultivating creative and individual designers. More challenging is the American architect, engineer, and futurist Richard Buckminster Fuller put forward the concept of “design scientist.” He believes that a “design scientist should be a generalist who can fully consider the production and Social connotation, not just considering the design of the product itself (1965).” In the book “Design Revolution: Earthship Spacecraft Operation Manual,” it is proposed that “synthesis” is a nature of mankind, which is different from all other creatures. It opposes excessive “specialization” and uses system theory and Synergy effects to talk about human development. “All other creatures on the earth have a highly specialized nature, with the exception of human beings. Human beings have a uniquely comprehensive understanding of understanding and coordinating affairs in a certain field. If the Creator’s intention was to make humans become an expert, then he should let humans have eyes with microscopes (1969).” In terms of specific teaching methods, with the application of AI technology in the education field, the traditional one-to-many teaching model can be changed, and new teaching methods such as one-to-one online teaching can be tried (Lei and Guochun, 2019). Especially in the face of extreme environments, such as the coronavirus disease 2019 (COVID-19) pandemic from 2020 to the present, the importance of changing the teaching mode and adopting online teaching methods has been highlighted (Pierson, 2001; Komis et al., 2007; Morsink et al., 2011; Kabakşı-Yurdakul et al., 2012; Rienties et al., 2013; Kirkland, 2014; Oakley and Pegrum, 2015; Hilton, 2016; Kihoza et al., 2016; Shah et al., 2017; Shadiev, 2018; Kemp, 2019; Seifi et al., 2020; Hsueh et al., 2021; Hughes et al., 2021; Jaspers, 2021; Özgen et al., 2021).

## Research Methodology and Research Process

**FIGURE 1:**
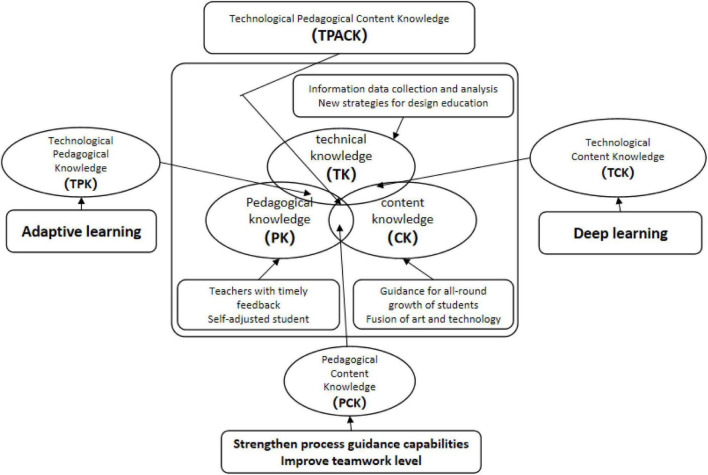
New strategies for designing education under the background of artificial intelligence technology.

With the development of technology, especially the widespread application of AI in the field of education, people have gradually realized that as a technological science, AI will affect the concept of talent training, innovate university teaching concepts and methods, and have an impact on the traditional education environment. Therefore, technology integration has gradually become a way to analyze the education process and solve educational problems. In line with this, some classic technology integration models have been formed from this. In America, Mishra and Koehler (2006) introduced the subject teaching knowledge model, TPACK (Technological Pedagogical Content Knowledge), integrated technology proposed the widest application and the greatest impact. In the context of AI technology, discussions and experiments take the TPACK (Figure 1) technology integration model, a path test, as the starting point to design education strategy research (Shulman, 1986; Shulman, 1987; Trigwell et al., 1999; Tawil and Locatelli, 2015; Tuncer and Bahadır, 2016; Tondeur et al., 2017; Soyupak and Bagli, 2019).

The specific method is based on the traditional design studio as the center, integrating the TPACK technology integration model, building an online design studio for teaching, and combining design project practice. Design studios have long proven to be an effective way to carry out design teaching. Because design education has its own unique attributes, the parallel teaching of design practice and theoretical knowledge will have a good tacit understanding. In addition, the design studio is also closer to the actual form of design work. Polanyi (2009), the creator of the studio concept, believes that certain types of knowledge cannot be transmitted or expressed through oral communication. In a similar way, Uluoğlu (2000) explained that people will only learn design through explanation if it is just to obtain a specific technology. In addition, the design studio provides students with a realistic experience by introducing hypothetical design issues to students (Tovey, 2015). Although the advantages of the studio are obvious, it is not possible to learn and practice in the same environmental space in the face of some extreme situations such as COVID-19. Also, under the rapid development of technology and technology, the design is constantly integrated to produce new types, new styles, and new branches. Design learning needs to be cross-professional, multi-disciplinary, and other issues. Research through the establishment of an online design studio project, with a new position assisted by AI technology, was used to raise questions and discuss from multiple angles, such as the following questions:

• How to implement design teaching based on the online format?
• How to ensure that students master design-related terms?
• How to import specific design project practices, and how to adapt different types of projects to the same process?
• How to establish specific learning needs through the direction of interest, and personalize learning content based on this?
• Extreme social cases and environments, such as COVID-19?
• What is the basis for online design studios to replace traditional teaching, and whether there are related sharing and conflicting effects with other commercial platforms?

To solve these problems, online scene construction is adopted for making clear learning plans according to the students’ interests of themselves, whilst customized learning content and design practice projects are applied as learning resource and guidance for improving their teamwork efficiency of joint design. With traditional design work on site other than deductive learning indoors involved, the students are supposed to integrate concept and knowledge of design, and form a good habit of self-driven learning as well. From theory to design practice, to operational practice, a relatively complete design professional education system is formed, thereby forming a set of comprehensive design teaching education strategies and deep learning methods in design education.

**FIGURE 2:**
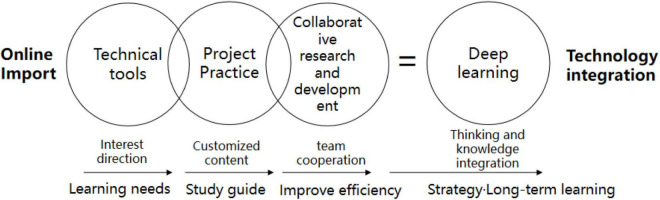
Implementation integration of online design studio.

We selected 40 students from the first year of animation design major and chose comprehensive evaluation as a method to identify and synthesize future design education trends from different types of research (knowledge concepts, design thinking, design concept system shaping, knowledge, and thinking transformation, analysis and comparison of project process planning, project implementation, teamwork, and other aspects). Combined with 10 specific design projects, the project was used to conduct a comprehensive analysis of problem identification, document retrieval, data evaluation, data analysis, design ideas, methods, paths, and tools. The following is an example of a specific design project:

In the implementation of the project, the integration themes carried out by the online design studio are as follows (Figure 2):

• Basic design education methods.
• Early design practice experience and interest integration.
• Facing the commercial needs of the front line of society.
• Adaptive learning of advanced technology driven by students.

**FIGURE 3:**
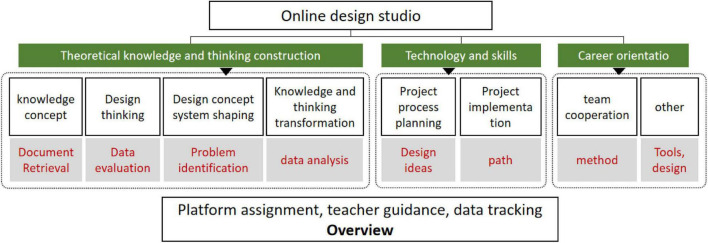
Design project process import.

**FIGURE 4:**
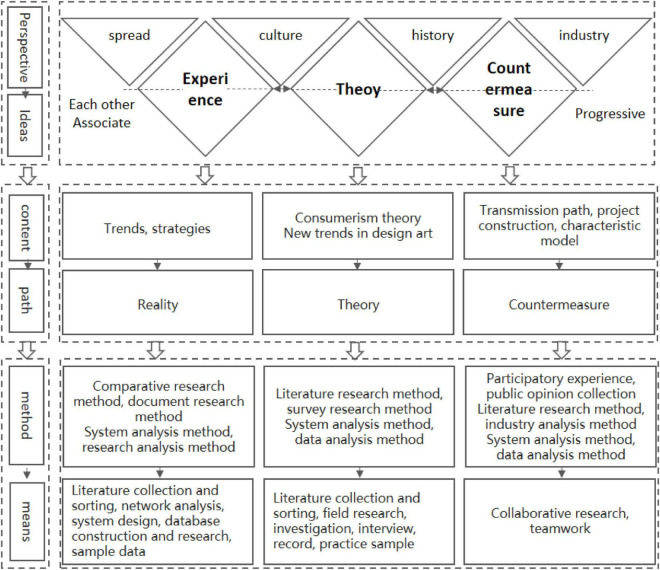
Design project implementation details.

At the level of the implementation, details of the project design, the perspective, ideas, content, paths, and methods are gradually advanced (shown in Figures 3, 4 for the implementation details of the project design). To construct a broad model. With the integration of data and technology, even if AI does not understand aesthetics, it can also adopt the formulaic (standardized) design. This is through the use of regular models and restricted parameters that are mass-produced, including more elements such as history, culture, environment, emotions, etc. This integration can also carry out the perfect interdisciplinary and comprehensive design. The solution can evolve based on such a model, redefine the new standard of the problem, and help generate new design education strategies and practices.

## Results

Forty students used the online design studio to carry out 10 practical design projects. Seven of the design projects were basic and three were comprehensive. Through the evaluation of six enterprise experts, the quality of eight studies was rated as “good” and the quality of two studies was rated as “fair.”

The research results show that the new online design studio education strategy is adopted. Students locate their learning needs from the direction of interest and conduct customized content learning, including choosing different design projects for practice, from theory to project implementation and completion. With personalized learning guidance, students’ learning needs show a better trend. In addition, customized educational content can enhance students’ learning experience and performance. For the cultivation of students’ design thinking, the establishment of learning interest directions and goals, the cultivation of thinking, and knowledge integration capabilities have a good improvement and long-term learning effects. In a highly open online format, each student could view the learning and project implementation of different teams. In the project, students start to learn adaptively, choose customized content with high learning interest, and can collaborate in a team for fruitful discussions.

## Discussion

Early educational experience and vertical integration are very helpful to promote effective lifelong learning, but in future design education, we should focus on strengthening the learning of art and technology and teamwork. In the specific design and learning of students, it is necessary to emphasize the collaboration between humans and AI. This study uses the TPACK model to conduct original experiments and conduct new technical docking on the basis of traditional design education. At the same time, the application and discussion are diversified, which makes the connection between students and specific commercial projects more convenient. The traditional design education courses which have established and implemented similar educational strategies, may be fruitful.

## Conclusion

The difference between AI and other digital technologies is that they can greatly reduce the cost and time of design and development. In this research, we explored online design studio education and opened a window of design education strategies. This research shows that online design studios built using AI technology have not undermined the principles and results of education. On the contrary, it further develops educational strategies by obscuring the traditional limitations of scale, environment, time, and collaboration. To achieve a human-oriented form of education, it can provide personalized learning guidance for everyone, provide customized educational content, and continue to improve as needed. It can also analyze user data according to personal circumstances, improve students’ learning experience and performance, and enhance creativity by expanding the scope of design practice. However, the development of design education in online design studios breaks the limitations of time and space and is completely different from previous education methods. We introduce TPACK to discuss this transformation and help capture the nature of education under the new AI technology. With the addition of AI technology, educational issues, especially educational data mining and student performance prediction, have been transferred to algorithms. Through deep learning, we can continue to innovate and find better solutions.

In terms of team cooperation, the online design studio center plan enables students to better cultivate team spirit, complete design project practice through online collaboration, and use high-tech for personalized learning, social interaction, and access to a large number of design resources. The online design studio can also expand design horizons more conveniently and effectively improve design efficiency. The application of AI technology is not to replace human teams, but to emphasize the collaborative relationship between humans and AI, and to create new ways of cooperation between AI and human teams.

## Suggestions for Further Studies

When AI technology began to change our traditional design education, the design studio was created as an online open learning platform where students had the opportunity to express their views freely and exchange ideas with different teams. In exploring new strategies and practices of design education, future studies could explore how to use a certain teaching format to encourage them to share and express ideas. In future studies, they can also collect feedback by adding more time and data. The scope of analysis can be further expanded by collecting responses of students to this stage of the project as qualitative and quantitative data, including more different types of cases, and conducting more comprehensive research, including situations similar to the COVID-19 pandemic. Future research may also discuss more of how to deal with the platform expansion of design learning of students and how to apply the project in practice so that students can fully grasp the time of project design and be more creative.

## Data Availability Statement

The raw data supporting the conclusions of this article will be made available by the authors, without undue reservation.

## Ethics Statement

Ethical review and approval was not required for the study on human participants in accordance with the local legislation and institutional requirements. The patients/participants provided their written informed consent to participate in this study.

## Author Contributions

PL: software. TT: conceptualization, methodology, data curation, and writing—original draft preparation. QT: visualization and investigation. All authors contributed to the article and approved the submitted version.

## Conflict of Interest

The authors declare that the research was conducted in the absence of any commercial or financial relationships that could be construed as a potential conflict of interest.

## Publisher’s Note

All claims expressed in this article are solely those of the authors and do not necessarily represent those of their affiliated organizations, or those of the publisher, the editors and the reviewers. Any product that may be evaluated in this article, or claim that may be made by its manufacturer, is not guaranteed or endorsed by the publisher.
